# Infection Complications in Hematopoietic Stem Cells Transplant Recipients: Do Genetics Really Matter?

**DOI:** 10.3389/fmicb.2018.02317

**Published:** 2018-10-02

**Authors:** J. Luis Espinoza, Yohei Wadasaki, Akiyoshi Takami

**Affiliations:** ^1^Department of Hematology and Rheumatology, Faculty of Medicine, Kindai University, Osaka, Japan; ^2^Division of Hematology, Department of Internal Medicine, School of Medicine, Aichi Medical University, Nagakute, Japan

**Keywords:** stem cells transplantation, GVHD, SNP, cytokine storm, microbiota, invasive infection

## Abstract

Hematopoietic stem cell transplantation (HSCT) is a highly advanced technique that offers a potential cure for an increasing number of life-threatening diseases. Enormous progress achieved in the last decade, including the refinement of donor selection and advancements in patient supportive care, had significantly improved transplant outcomes; however, invasive infections, graft-vs.-host disease (GVHD) and other serious complications still represent a major source of morbidity and mortality in HSCT recipients. The damage of anatomical barriers due to pre-transplant conditioning, a severely damaged immune function and a profound disruption in the composition of gut microbial commensals (gut microbiota) are alterations inherent to the transplant procedure that are directly implicated in the development of invasive infections and other HSCT complications. Although HLA-matching represents the most important genetic predictor of transplant outcomes, genetic variants in non-HLA genes, especially single nucleotide polymorphisms (SNPs) of genes encoding proteins associated with the immune response to tissue injury and pathogen infection have also been proposed as additional risk factors implicated in the occurrence of HSCT complications. Furthermore, although the microbiota composition is affected by several factors, recent evidence suggests that certain host genetic variants are associated with an altered composition of the gut microbiome and may, therefore, predispose some individuals to invasive infectious complications. This article summarizes the current understanding of the influence that genetic variants in non-HLA genes have on the development of infectious complications in HSCT recipients.

## Introduction

Hematopoietic stem cell transplantation (HSCTs) involves the intravenous infusion of hematopoietic stem and progenitor cells (HSPCs) to restore the bone marrow (BM) function after intensive cancer-eradicating treatments or to replace defective bone marrow with normal bone marrow in non-malignant hematologic disorders (Gratwohl et al., 2010; Passweg et al., 2016). Currently, HSCT is utilized with curative intent in a variety of blood malignant diseases, such as leukemias, lymphomas and myeloma, as well as some solid tumors, such as germ cell tumors, soft tissue sarcomas and neuroblastoma (Holtick et al., 2014; Passweg et al., 2016) and is a curative option for thalassemia, sickle cell anemia, severe combined immunodeficiency and for young patients with severe aplastic anemia (Bacigalupo and Sica, 2016).

HSPCs for HSCT are harvested from BM, peripheral blood (after the donor receives granulocyte colony-stimulating factor to mobilize HSPCs) or cord blood, being donor availability, patient's age and the underlying disease the main factors that determine the decision to use a certain source of HSPCs (Holtick et al., 2014; Niederwieser et al., 2016). Although BM was the main source of HSPCs for many years, currently HSCT is most frequently done with peripheral blood stem cells (PBSCT) (Gratwohl et al., 2010; Anasetti et al., 2012; Juric et al., 2016).

Depending on the donor source, HSCT can be autologous (auto-HSCT) or allogenic (allo-HSCT) (Anasetti et al., 2012; Juric et al., 2016). Auto-HSCT is essentially indicated in patients undergoing intensive or high-dose chemoradiotherapy (myeloablative) for the treatment of certain malignant diseases and involves the harvesting and cryopreservation of HSPCs from patients themselves and then reinfusing those HSPCs into the patients after myeloablative therapy in order to restore hematopoiesis and hasten the immune function (Niederwieser et al., 2016; Bogunia-Kubik and Łacina, 2017). Allo-HSCT is the transplantation of HSPCs from a histocompatible healthy donor that may be a fully matched or half matched (haploidentical) family relative to the patient or an unrelated donor (Juric et al., 2016; Niederwieser et al., 2016).

A critical component of HSCT is the conditioning regimen administered prior to the infusion of HSPCs and is aimed to eradicate the patient's disease and to prevent rejection of the transplanted graft. Conditioning can be myeloablative, in which the recipient BM is destroyed (ablated), usually using high doses of chemotherapy and whole-body irradiation, or non-myeloablative (Holtick et al., 2014; Juric et al., 2016). Graft-vs.-host disease (GVHD) and transplant-related mortality (TRM) constitute important limitations associated with HSCT (Gratwohl et al., 2010; Passweg et al., 2016). In addition, the exposure of HSCT recipients to multiple antibiotics, utilized either for curative or preventive purposes, eventually results in the disruption of the gut microbiota composition that further increases the patients' susceptibility to severe infection complications (Taur et al., 2014; Dandoy et al., 2017).

HLA-matching is the most important genetic determinant of transplant outcomes; however, single-nucleotide polymorphisms (SNPs) in non-HLA genes, especially in genes encoding immune response factors, appear to affect HSCT outcomes, including relapse, survival and GVHD, (Espinoza et al., 2009; Dickinson and Norden, 2015; Bogunia-Kubik and Łacina, 2017).

This article highlights potential associations between genetic variants in genes encoding non-classical HLA proteins and the development of infection-related complications in HSCT recipients. Recent studies that appear to link genetic variants in certain genes with specific changes in the gut microbiota composition are also discussed.

## Infectious complications in HSCT recipients

Conditioning regimens required for HSCT incite mucosal injuries and destroys hematopoiesis leading to the damage of normal anatomical barriers and extensive immunodeficiency, especially in those receiving myeloablative regimens (Balletto and Mikulska, 2015; Zama et al., 2017). As a result, infection complications are major cause of morbidity and mortality in HSCT recipients and are often associated with commensal bacteria and fungi derived from the gastrointestinal tract and with opportunistic organisms colonizing the skin that entry the bloodstream via intravenous catheters. Engraftment, which occurs at a range: 6–84 days after HSCT (median 22 days), is defined by the capacity of HSCT recipients to maintain an absolute neutrophil count of > 500/mm^3^ and platelet count of >20,000, lasting >3 consecutive days without transfusions. The median time to engraftment is longer for bone marrow transplantation (BMT) recipients than in those receiving PBSCT, which may explain the relative higher risk of infections in BMT in comparison with PBSCT (Young et al., 2016).

Immune reconstitution after HSCT occurs in three phases: the pre-engraftment phase (<30 days after HSCT); early post-engraftment phase (30–100 days after HSCT); and late post-engraftment phase (>100 days after HSCT) (van den Brink et al., 2015; Ogonek et al., 2016). Each phase is characterized by specific host-defense defects with defined risk of infections (Table 1). During the pre-engraftment phase the presence of neutropenia, severe mucositis and central venous catheter are major risks of infection (Gudiol et al., 2014). The majority of infections in this period are due to bacteria, with Gram positive organisms predominating over Gram negatives (Gudiol et al., 2014; Kikuchi et al., 2015; Schuster et al., 2017; Mikulska et al., 2018). *Clostridium difficile* infection in HSCT recipients most often occurs during early pre-engraftment (Schuster et al., 2017) and fungal infections (*Candida* spp. and *Aspergillus* spp.) and herpes simplex virus (HSV) reactivation can be also seen in this phase (Balletto and Mikulska, 2015; Sahin et al., 2016; Alp and Akova, 2017; Cho et al., 2018).

**Table 1:** Infections complications in Hematopoietic stem cells transplantation recipients.

**Type of infection**	**Early pre-engraftment (2-4 weeks after HSCT)**	**Early post-engraftment (2-3 months after HSCT)**	**Late phase (beyond 3 months after HSCT)**
Bacterial (Gudiol et al., 2014; Kikuchi et al., 2015; Young et al., 2016; Girmenia et al., 2017; Schuster et al., 2017; Cho et al., 2018; Mikulska et al., 2018)	Gram positive bacteria (Coagulase-negative *staphylococci, streptococcus pneumoniae, viridans group streptococci, staphylococcus aureus*) Gram negative bacteria (*Escherichia coli, Klebsiella pneumoniae, Pseudomonas aeruginosa*) *Clostridium difficile*	Gram positive bacteria (*Streptococcus pneumoniae* Coagulase-negative *staphylococci, viridans group streptococci, staphylococcus aureus*) Gram negative bacteria (*Escherichia coli, Klebsiella pneumoniae, Pseudomonas aeruginosa*)	Encapsulated bacteria (mainly *streptococcus pneumoniae and Haemophilus influenza*) *Pseudomonas aeruginosa* *Nocardia*
Fungal(Gudiol et al., 2014; Kikuchi et al., 2015; Sahin et al., 2016; Young et al., 2016; Maertens et al., 2018)	*Candida species*. *Aspergillus species*.	*Aspergillus species* *Pneumocystis jirovecii*	*Aspergillus species* *Pneumocystis jirovecii*
ViralLin and Liu, 2013; Sahin et al., 2016; Cho et al., 2018	HSV (mostly reactivation of HSV latent infection)	Mainly CMV. Community acquired viruses (mainly respiratory viruses) Other herpes viruses (EBV, HHV-6) *Polyoma virus*	CMV VZV EBV (risk of PTLD) Community acquired viruses (mainly respiratory viruses)
Parasitic	Rarely documented	Rarely documented Toxoplasma gondii Jarque et al., 2016; Prestes et al., 2018 (usually reactivation of a latent infection). **Other parasites:** Jarque et al., 2016; Fabiani et al., 2017 Reactivation of latent infection or transmission from infected donors: Especially in endemic regions (*Tripanozoma cruzy, Plasmodium spp. Leishmania spp*)	Rarely documented Toxoplasma gondii Jarque et al., 2016; Prestes et al., 2018 (usually reactivation of a latent infection) **Other parasites:** Jarque et al., 2016; Fabiani et al., 2017 Reactivation of latent infection or transmission from infected donors: Especially in endemic regions (*Tripanozoma cruzy, Plasmodium spp. Leishmania spp*) Primary infection with other parasites rare and may have worldwide distribution (*Cryptosporidium spp, Blastocystis spp*.) or limited to endemic regions: *Strongyloides stercoralis*

During the post-engraftment phase, the weakened cell-mediated immunity, together with the development of GVHD and the use of corticosteroids and immunosuppressive constitute key risk factors of infection, especially pneumonia and gastrointestinal infections. In this phase, Gram positive organisms also predominating over Gram negatives (Gudiol et al., 2014; Sahin et al., 2016; Cho et al., 2018). In the late post-engraftment phase patients have increased risk for infections with encapsulated bacteria, due to hypogammaglobulinemia and The exposure to immunosuppressive treatment for controlling GVHD. In addition, Invasive fungal infections, cytomegalovirus (CMV), varicella-zoster virus (VZV), EBV-related post-transplant lymphoproliferative disease (PTLD) can be seen during this period.

## SNPs of immunoregulatory genes and HSCT

The most frequent genetic variations of the human genome are SNPs, and the majority occur in non-coding regions, including intronic, intergenic, and untranslated regions (UTRs). Some are considered functional, as they can affect the gene expression or may alter the function of their coding protein (Dickinson and Norden, 2015; Bogunia-Kubik and Łacina, 2017). To identify non-HLA gene SNPs associated with transplant outcomes, two approaches have been utilized so far: candidate gene studies (CGSs), which directly test target gene variants selected on the basis of the biological potential reported in previous studies; and genome-wide association studies (GWASs), in which many gene polymorphisms are examined on a hypothesis-free basis. With both the CGS and GWAS approaches, it is important that the association found in a discovery cohort also be observed in an independent cohort in order to validate the association (Takami, 2013; Bogunia-Kubik and Łacina, 2017).

For over a decade, several groups, including our own, have reported that genetic variants outside of the HLA region, especially in genes encoding components of the immune system, are predictive of the outcome following HSCT (Espinoza et al., 2009; Takami, 2013; Bogunia-Kubik and Łacina, 2017). However, many of these gene-association studies were conducted in small series, and some included heterogeneous diseases with patients treated across multiple years with variable transplant regimens; furthermore, the data were often not adjusted to a standardized format (Karaesmen et al., 2017). In addition, the majority of the published studies did not include a confirmatory cohort, so few of the reported gene associations have been consistently validated. Nonetheless, a number of polymorphisms in genes encoding cytokines and chemokines, such as INF-γ, IL-2, IL-1, IL-6, and TNF-α, Granzyme B (GZMB), IL-17 and their receptors, have been reported in association with various transplant outcomes, including the overall survival, disease relapse, graft rejection and GVHD (Espinoza et al., 2009, 2011a,b; Nakata et al., 2013; Dickinson and Norden, 2015; Bogunia-Kubik and Łacina, 2017; Gam et al., 2017).

## Non-HLA variants and infectious complications after HSCT

Early studies showed that the donor and recipient genotypes in genes that regulate the host response to microorganisms, including myeloperoxidase (MPO), mannose binding lectin (MBL) and Fcγ receptors (FcγRIIa, IIIa, IIIb), were reportedly associated with infections after BMT (Rocha et al., 2002). In addition, the steroid hormone receptor supergene family includes the estrogen receptor and the vitamin D receptor (VDR), both of which have marked effects on the development of the immune system (Rocha et al., 2002; Middleton et al., 2003). The AA genotype of the VDR gene in recipients is associated with less severe GVHD, while its presence in the donor appears to lead to a reduced protective effect against infection and relapse, most notably in those treated with more intense immunosuppressive therapies (Middleton et al., 2002).

Mezger et al. reported an association between the recipient CXCL10 and the development of invasive aspergillosis in a European cohort of 81 allo-HSCT recipients (Mezger et al., 2008). The variant 642C/G, which is determined by the rs3921 SNP, has been associated with susceptibility to other infections (Pineda-Tenor et al., 2014; Hoh et al., 2015). In a larger cohort of 652 HSCT Japanese patients who underwent unrelated HLA-matched (BMT) for hematologic malignancies, our group found that a recipient C/G or G/G genotype associated with a significantly better 5-year overall survival (OS) rate, a lower TRM rate and a reduced incidence of death due to organ failure compared with the recipient C/C genotype (Nakata et al., 2013). CXCL10 is produced by innate immune cells and by several non-hematopoietic cells, including endothelial cells, vascular pericytes, keratinocytes, fibroblasts and astrocytes, and is considered a “key driver chemokine” that has been linked with the pathogenesis of autoimmune diseases, such as inflammatory bowel disease (IBD), multiple sclerosis and rheumatoid arthritis, and is involved in the immune response to infections (Karin and Razon, 2018). Elevated levels of CXCL10 have been consistently observed in bodily fluids and inflamed tissues of HSCT recipients with acute GVHD, so this chemokine has been proposed as a biomarker of this transplant complication (Ahmed et al., 2015).

Toll-like receptors (TLRs) are a family of transmembrane proteins on the surface of immune cells that detect microbe-associated molecular patterns (MAMP) from a variety of microorganisms. In response to their interaction with microbial motifs, TLRs activate intracellular signals, leading to the synthesis of cytokines and other inflammatory mediators. Although TLRs constitute the first line of defense against many pathogens, they may also promote excessive immune responses, leading to tissue damage. Previous studies have implicated TLR signals in cytokine storm and the inflammatory process leading to GVHD and other complications in HSCT recipients (Maeda, 2013; Heidegger et al., 2014), and several SNPs in *TLR* genes have been associated with susceptibility to infections (Skevaki et al., 2015; Dhangadamajhi et al., 2017). Bochud et al. reported that donor *TLR4* haplotype S4, which is defined by the *TLR4* variations rs4986790 [D299G] and rs4986791 [T399I], was associated with a risk of invasive aspergillosis after allo-HSCT (Bochud et al., 2008). This haplotype appears to influence the TLR4 function, but it is absent in Asian populations (Aki et al., 2015). In a cohort of 365 Japanese patients who underwent unrelated HLA-matched bone marrow transplantation (BMT) for hematologic malignancies, the SNP rs11536889 in *TLR4* gene, whose minor allele frequencies in the Asian population are relatively high (>0.2) (Tahara et al., 2010; Sato et al., 2012), +3725G/G genotype in the donor side was significantly associated with a better 5-years progression-free survival and a lower 5-years TRM than other variations. Furthermore, the donor TLR4 genotype +3725G/G was associated with a significantly lower incidence of fatal infections than other variations and these results were confirmed in a validation cohort (Uchino et al., 2017).

Another component of the innate system involved in the response to pathogen products is the nucleotide-binding oligomerization domain-containing protein 2 (NOD2). This intracellular sensor recognizes conserved motifs in bacterial peptidoglycan and activates intracellular signals that drive antimicrobial immune responses cooperating with TLRs in the detection and elimination of invading microbes (Caruso et al., 2014). NOD2 is considered a major risk factor for the development of IBD (Caruso et al., 2014). SNPs within the *NOD2* gene are associated with the susceptibility to various bacterial infections (van Well et al., 2013; Branković et al., 2015) and have been linked with the risk for allo-HSCT complications, including TRM, GVHD, and bronchiolitis obliterans (Holler et al., 2004; Hildebrandt et al., 2008). In a small cohort of allo-HSCT recipients, the donor *NOD2-*SNP13 was significantly associated with the incidence of septic shock (Grube et al., 2015). The association between the *NOD2-*SNP13 and the incidence of sepsis in the context of allo-HSCT was further replicated in a larger cohort (464 patients) of allo-HSCT recipients treated in five different transplantation units in Poland (Jaskula et al., 2014), thus substantiating the biological significance of this genetic variation in infectious complications.

Dectin-1 is a C-type lectin receptor member expressed on the surface granulocyte immune cells that plays an important role in the immune response to fungal pathogens by sensing the cell wall components of fungi, including *Candida* species and *Aspergillus* species (Hirose et al., 2017). In a cohort of 142 patients undergoing HSCT, recipients harboring the Y238X polymorphism in the *DECTIN-1* gene were more likely to be colonized with *Candida* species than patients bearing WT *DECTIN-1* and required the more frequent use of fluconazole to prevent systemic *Candida* infection (Plantinga et al., 2009). In a cohort of 205 patients receiving allo-HSCT for blood malignancies, the presence of the DECTIN1 Y238X polymorphism was found to be associated with an increased susceptibility to aspergillosis, with the risk being highest when Y238X was present simultaneously in both donors and recipients (adjusted hazard ratio = 3.9; *P* = 0.005) (Cunha et al., 2010). Functional studies showed a decreased cytokine production by immune cells bearing this polymorphism on fungal-specific stimulation (Plantinga et al., 2009; Cunha et al., 2010). Dectin-1 knockout (KO) mice showed a higher fungal burden than wild type mice indicating that Dectin-1 is crucial for clearing fungal infections (Cunha et al., 2010).

## The influence of human microbiota on the outcomes of transplantation

Antibiotics that destroy gut commensal microbiota communities deplete the host's commensal gut bacteria and ultimately allow the growth of pathogenic microbial species (Zama et al., 2017), and the outgrowth of opportunistic pathogens belonging to the phylum *Proteobacteria* and genus *Enterococcus* have also been linked to increased TRM, including GVHD, infections and organ failure after allo-HSCT (Staffas et al., 2017).

**Figure 1:**
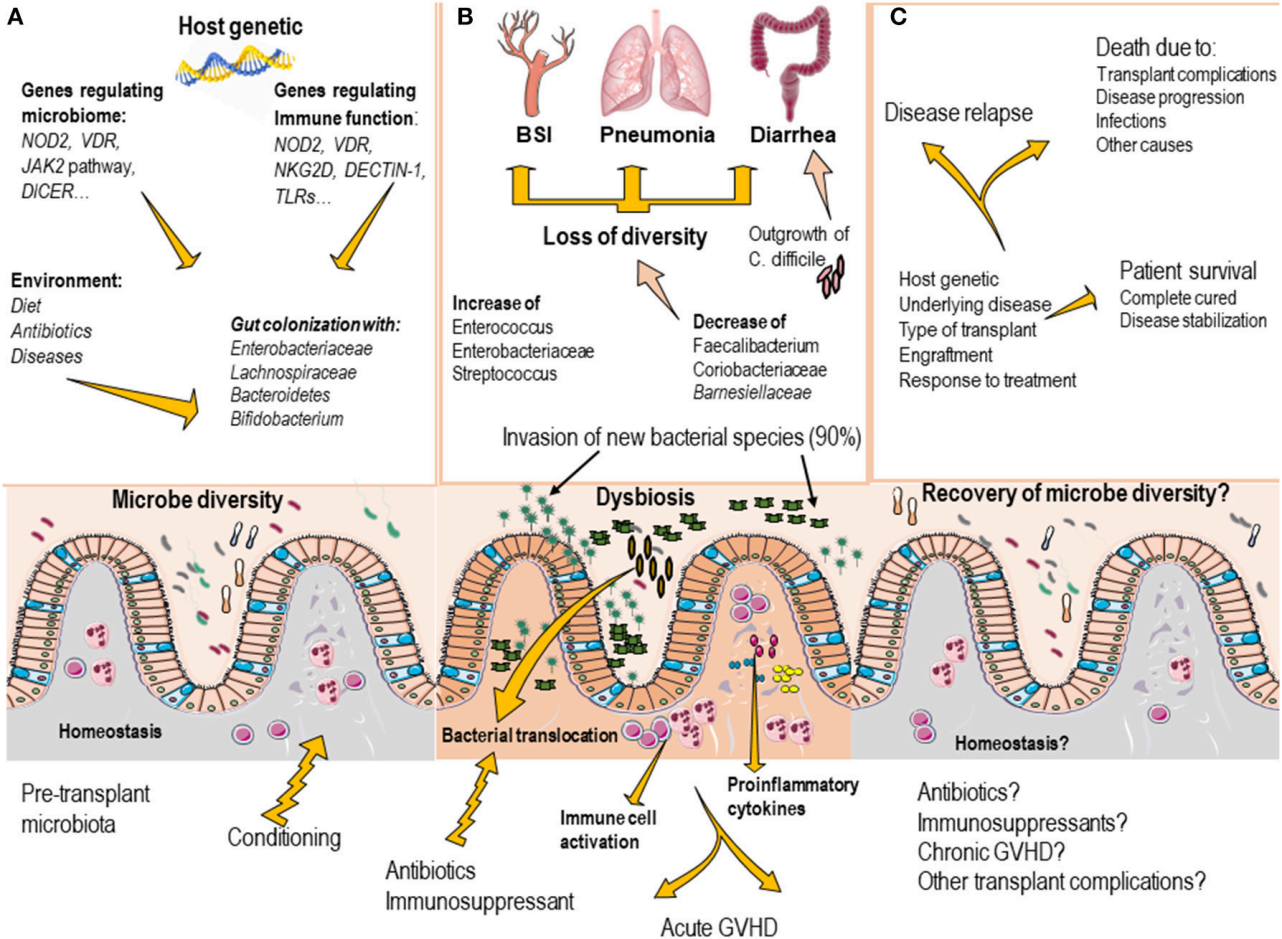
Commensal bacteria of the gut are important modulators of tissue and immune homeostasis **(A)**. The gut microbiota composition is mainly regulated by environmental factors, including diet, use of antibiotics, and the disease status. Host genetics factors appears to contribute to regulate the gut microbiota composition as genetic variations in genes that regulate immune response and genes that directly influence microbiota has been identified. **(B)** In transplant recipients, the conditioning regimen and the use of antibiotics, induce profound disruption of gut microbiota composition (loss of bacterial diversity, decrease of protective species and outgrowth of pathogenic microorganism), which may result in the development of invasive infections (BSI, pneumonia and *clostridium difficile*–induced diarrhea). Dysbiosis also contributes to increase the risk to develop transplant complications, such as graft vs. host disease. **(C)** As the transplant progress, most patients recover the gut microbiota composition (usually by day 60 after transplant), however the presence of transplant complications, massive use of antibiotics may delay the gut microbiota recovery.

Compelling evidence suggest that microbiota composition before transplant may predict the risk of infection complications, including bloodstream infections (BSI) (Figure 1). For example, patients who develop BSI after HSCT, exhibit decreased microbiome diversity and decreased abundance of taxa including *Barnesiellaceae, Coriobacteriaceae, Faecalibacterium, Dehalobacterium, and Sutterella* (Montassier et al., 2016). In pediatric allo-HSCT recipients, acute-GVHD and non-acute-GVHD subjects also possess a different pre-HSCT gut microbiota composition. In particular, non- acute-GVHD patients showed a pre-HSCT gut microbiota characterized by a higher abundance of members of the Bacteroidetes phylum (*Parabacteroides* and *Bacteroides*) (Biagi et al., 2015).

In other study, the presence of *Enterobacteriaceae* at the beginning of the pretransplant conditioning regimen in stool samples of allo-HSCT recipients was the only significant marker for the risk of microbiologically confirmed sepsis. An increased risk of overall mortality, including death from both infectious and non-infectious causes, was observed when *Lachnospiraceae* were ≤10% at the beginning of the pretransplant conditioning regimen (Mancini et al., 2017). In addition, the presence of 3 distinct bacterial taxa (*Bacteroidetes, Lachnospiraceae*, and *Ruminococcaceae*) in the gut microbiome of allo-HSCT recipients correlated with protection against *C. difficile* infection (Lee et al., 2017). These data strongly suggest that manipulating the gut microbiota may be useful to prevent BSI in high-risk patients and efforts aimed at maintaining protective commensals may help to minimize the risk of *C. difficile* infection in allo-HSCT recipients.

## Genetic variation and microbiota composition

Environmental factors play a central role in determining the composition of human microbiota; however, accumulating evidence suggest that host genetics can influence the microbiome composition. The host genetics regulate the immune system, nutrient availability and the secretion of immunoglobulin A (IgA) and thus can shape the chemical and physical composition of the environment inhabited by the gut microbiome.

Recent microbiome genome-wide association studies (mGWASs), which are cohort studies that combine human genotyping or GWASs with microbiome analyses, have identified numerous associations between the gut microbiome and specific human genetic variants (Table 2) (Goodrich et al., 2014, 2016; Knights et al., 2014; Wacklin et al., 2014; Davenport et al., 2015, 2016; Beaumont et al., 2016; Bonder et al., 2016; Turpin et al., 2016; Wang et al., 2016). Thus far, the most consistent evidence emerging from mGWAS that support a role for the host genetics in defining the composition of the gut microbiome is the link between the host genotype, the post-weaning consumption of milk and the colonization with *Bifidobacterium*, which has been consistently validated in various cohorts (Goodrich et al., 2017; Hall et al., 2017). In addition, several immunity-related risk alleles have been found to alter the microbiome composition. For example, Knights et al. reported that individuals carrying specific NOD2 variants show significant enrichment of *Enterobacteriaceae* bacteria (Knights et al., 2014). These findings may support the above discussed observations linking NOD2 variants with severe infectious complications in HSCT (Holler et al., 2004; Hildebrandt et al., 2008; Jaskula et al., 2014; Grube et al., 2015), as such infections in HSCT recipients are associated with gut microbiota dysbiosis (Zama et al., 2017). Of note, the study by Knights et al. also identified specific bacteria taxa in the gut microbiome associated with various IBD-related SNPs and involving genes that regulate the innate immune response and the JAK-STAT pathway (Knights et al., 2014).

**Table 2:** Studies investigating potential associations between human genetics and microbiota composition.

**Type of study**	**Number of subjects**	**Gene associations**	**Main findings**	**References**
mGWAS (twin pairs)	1,126	8.8% of taxa are heritable	Associations between host genetics and colonization with *Methanobrevibacter* and *Blautia*	Goodrich et al., 2016
mGWAS (twin pairs)	545	6% of taxa are heritable	Identified associations between host genetics and gut microbiome Some components associated with visceral fat	Beaumont et al., 2016
mGWAS (individuals)	1,812	42 SNPs associated with β diversity that together explained 10% of the variance of microbiome β-diversity	Human gut microbiome associated with VDR gene. Verified altered microbiome in VDR-deficient mice	Wang et al., 2016
mGWAS (twin pairs)	416	5.3% of taxa heritable	Some species of the gut microbiome are heritable, including the family *Christensenellaceae*	Goodrich et al., 2014
mGWAS (individuals) whole-metagenome sequencing	1,514	9 SNPs associated with microbial taxa, 33 SNPs associated with microbial pathways	LINGO2 variants associated with the genus *Blautia* and family *Methanobacteriaceae*	Bonder et al., 2016
mGWAS (individuals)	1,561	58 SNPs associated with 33 microbial taxa	Identified host gene variants associated with the abundance of *Rikenellaceae, Faecalibacterium, Lachnospira* and *Eubacterium*	Turpin et al., 2016
mGWAS (individuals)	474	49 SNPs associated with microbial taxa	Colonization with *Enterobacteriaceae* in NOD2-deficient individuals	Knights et al., 2014
mGWAS (individuals)	127	8 bacterial species associated with SNPs	Colonization with the genus *Akkermansia* and the gene PLD1 in a Hutterite population	Davenport et al., 2015
16S rRNA gene pyrosequencing + PCR-DGGE (individuals) mGWAS (twin pairs) mGWAS (individuals)	71 1,503 1,190	SNP rs601338 (FUT2) SNP rs601338 (FUT2) ABO secretor status SNP, rs601338 (FUT2) Dominant phyla: *Firmicutes, Bacteroidetes, Actinobacteria*.	rs601338 associated with secretor status and with human microbiota composition No association between FUT2 genotype with human fecal microbial composition and ABO secretor status No association between FUT2 with human fecal microbial composition and imputed function.	Wacklin et al., 2014 Davenport et al., 2016 Turpin et al., 2018
mGWAS (individuals) Measurements: Anthropometric Blood Dietary habits	1,046	Estimate heritability of gut microbiome taxa 1.9%	Significant similarities in the compositions of the microbiomes of genetically unrelated individuals who share a household. Over 20% of the inter-person microbiome variability is associated with factors related to diet, drugs and anthropometric measurements.	Rothschild et al., 2018

A recent GWAS of the gut microbiota using two cohorts from Germany (1,812 individuals in total) revealed significant associations between the overall microbial variation and individual taxa at multiple genetic loci, including the VDR gene. In addition, the microbiota composition correlated with the serum levels of selected bile and fatty acids, including known ligands and downstream metabolites of VDR (Wang et al., 2016). Mouse experiments conducted by the same team revealed significant shifts in the microbiota of Vdr^−/−^ mice relative to control mice (Wang et al., 2016), which is consistent with a previous study that observed the massive depletion of *Lactobacillus* with the concomitant enrichment of *Clostridium* and *Bacteroides* in the intestines of Vdr^−/−^ mice (Jin et al., 2015).

FUT2 encodes fucosyltransferase 2, a major component of mucosal barrier that serves as energy source for some bacteria and a binding site for others. A common nonsense mutation (428G > A) in the FUT2 gene determines that individuals who have an AA genotype lack terminal fucose in the mucin layer; these people are considered nonsecretors (Hall et al., 2017). GWAS identified a FUT2 variant (AA genotype) associated with an altered gut microbiome, FUT2 nonsecretor-status and increased risk of Crohn's disease (McGovern et al., 2010). The AA genotype has been also linked with increased susceptibility to certain infections (Lindesmith et al., 2003; Folseraas et al., 2012) and with increased incidence of bacteremia and high risk of acute GVHD in allo-HSCT recipients (Rayes et al., 2016). Notably, no association was found between FUT2 genotypes, microbiota composition and secretor status in two large cohorts of healthy, highlighting the importance of replicating microbiome-associated traits in large cohorts (Davenport et al., 2016; Turpin et al., 2018).

Pathogenic bacteria are capable of modulating the expression of small non-coding micro RNAs (miRNAs) in the host, and given the crucial role of miRNA in the post-transcriptional regulation of several host genes, bacterial can thus manipulate host cell pathways that are directly implicated in the outcome of infection (Maudet et al., 2014). However, emerging evidence suggests that commensal microorganisms are also capable of regulating the host miRNA expression (Williams et al., 2017). Liu et al. recently reported that miRNAs are detectable in the gut lumen and feces of both mice and humans and that these RNAs are able to regulate the gut microbiota composition (Liu et al., 2016) and Moloney et al, proposed that the fecal miRNA composition can be used as an independent biomarker of microbial fluctuations along with the gut pathology in the intestine (Moloney et al., 2018). It must be noted that SNPs affecting the miRNA levels, such as those implicated in miRNA biogenesis or SNPs capable of affecting the miRNA/target interaction, can modulate the expression of cytokines, chemokines or immune receptors encoding genes and consequently may contribute to the risk of complications associated with HSCT (Gam et al., 2017; Stickel et al., 2017). For example, the SNP rs1049174 in the KLRK1 gene (which encodes the immune receptor NKG2D) resides in a motif that is targeted by miRNA1245, a negative regulator of the NKG2D expression. Notably, allo-HSCT recipients receiving grafts from donors with the LNK genotype of rs1049174 have worse transplant outcomes, including an impaired OS and increased risk of TRM, than recipients receiving graft from donors with the HNK genotype in rs1049174(Hayashi et al., 2006; Espinoza et al., 2009). Natural killer (NK) cells from individuals harboring the LNK genotype express lower levels of NKG2D on their surface and have lower NKG2D-mediated functions, including killing target cells expressing NKG2D ligands on their surface than NK cells from HNK due to the higher affinity of the LNK allele for miR1245 (Espinoza et al., 2012, 2016). Consequently, rs1049174 is implicated in HSCT outcomes via the regulation of the NKG2D expression by immune cells.

## Concluding remarks and future directions

HSCT recipients often suffer severe alterations in their microbiota composition, and it has become evident that dysbiosis, especially that of the gut microbiota, is involved in the development of transplant complications, including acute GVHD and invasive BSI.

Although the human microbiota is essentially determined by environmental factors, convincing evidence indicates that genetics help regulate the microbiota composition. Of particular interest is the finding that several immunity-related risk alleles, such as NOD2 variants, have been shown to alter the microbiome composition in association with *Enterobacteriaceae* family enrichment in the gut microbiota (Knights et al., 2014), which may explain the link between NOD2 variants and severe infectious complications in HSCT (Holler et al., 2004; Hildebrandt et al., 2008; Jaskula et al., 2014; Grube et al., 2015). However, most previously reported associations failed to reach statistically significance after multiple testing correction (Kurilshikov et al., 2017) and a recent study that evaluated a cohort of several different ancestral origins that share a relatively homogeneous environment demonstrate that gut microbiome composition is shaped predominantly by environmental factors (Rothschild et al., 2018).

Fecal microbiota transplantation (FMT), which consists of the infusion of fecal material or fecal bacterial preparations from a healthy donor into the gut of a recipient patient with altered microbiota, is safe and effective in controlling *C. difficile* infection after HSCT (Webb et al., 2016). This indicates that therapeutic interventions aimed at modifying the altered microbiota, such as FMT or via the use of probiotics, may have clinical utility in certain patients. Alternative approaches may include the use of more precise antibiotics as well as dietary changes, nutritional prebiotic supplements that support the growth of commensal organisms, probiotics and FMT. In addition, the use of “engineered microbiota,” has been shown in preclinical models to have great therapeutic potential. For example, a mixture of comprehensively selected strains of *Clostridia* was very effective in promoting the expansion and differentiation of Tregs in a mouse model of colitis (Atarashi et al., 2013), and the precision reconstitution of *C. scindens*, a commensal that confers resistance to *C. difficile* colonization via bile acid production, was very effective in preventing *C. difficile* infection (Buffie et al., 2015). Finally, the use of artificial intelligence tools capable of integrating clinical information with microbiome data represent a promising strategy for tackling infection complications in HSCT recipients (Espinoza, 2018).

## Author contributions

JE conceived the study, wrote the manuscript, and designed figures. YW collected references and wrote the manuscript. AT wrote the manuscript.

### Conflict of interest statement

The authors declare that the research was conducted in the absence of any commercial or financial relationships that could be construed as a potential conflict of interest.
